# Pathways between childhood trauma, clinical symptoms, and functioning in new-onset psychosis: novel insights from a network analysis approach

**DOI:** 10.1038/s41537-025-00620-2

**Published:** 2025-05-15

**Authors:** Dimitrios Kiakos, Luis Alameda, Ines Lepreux, Caroline Conchon, Livia Alerci, Marianna Gorgellino, Nadir Mebdouhi, Teya Petrova, Philippe Golay, Lilith Abrahamyan Empson, Philippe Conus, Sandra Vieira

**Affiliations:** 1 Service of General Psychiatry, Lausanne University Hospital (CHUV), Lausanne, Switzerland; 2 Department of Psychosis Studies, Institute of Psychiatry, Psychology and Neuroscience, King’s College, London, UK; 3 National Psychosis Unit, South London and Maudsley NHS Foundation Trust, London, UK; 4 La Source School of Nursing, HES-SO University of Applied Sciences and Arts of Western Switzerland, Lausanne, Switzerland

**Keywords:** Psychosis, Schizophrenia

## Abstract

Childhood trauma (CT) has been linked to various domains of outcomes in individuals with new-onset psychosis, but the intricate relationships between different types of trauma, clinical symptoms, and functioning remain poorly understood. The aim of this study was to identify patterns of relationships between these three domains in first-episode psychosis (FEP). The study sample consisted of 277 patients from the Treatment and Early Intervention in Psychosis Program (TIPP) at Lausanne University Hospital. Symptom severity was assessed using the Positive and Negative Syndrome Scale (PANSS), functioning was evaluated with an adapted version of the general subscale of the Premorbid Adjustment Scale (PAS), and the five most common CT experiences (sexual, physical and emotional abuse; physical and emotional neglect) was measured using a tailored questionnaire. Data from early assessments (2 and 6 months after admission) were used for analysis. A network model was fitted to estimate the shortest pathways linking different types of CT to various domains of functioning. Our findings revealed two distinct pathways connecting CT to functioning. One pathway influenced occupational functioning through sexual abuse and depression, while another pathway affected socio-personal functioning through physical neglect and stereotyped thinking. Our results suggest that distinct disease phenotypes could be differentially associated with CT and functioning in individuals with new-onset psychosis. This study contributes to the growing evidence supporting the existence of multiple distinct pathways to psychosis, each linked to a different clinical phenotype.

## Introduction

In the field of psychosis, childhood trauma (CT) refers to exposure to potentially traumatic events, such as sexual, physical, or emotional abuse, physical or emotional neglect, bullying, or early separation, occurring before the age of 16 or 18^1^. CT is a risk factor for psychosis^2^, and it can negatively affect the clinical profile and worsen outcome in those with a psychotic disorder (PD)^1,3–5^. One of the major negative consequences of CT in individuals with PD is the resulting impairments in functioning. Several studies investigating the impact of CT on functioning in schizophrenia^6,7^, first-episode psychosis^8^ (FEP), and ultra-high risk (UHR) for psychosis^9^ have found a significant association between early traumatic experiences and impaired functioning in adulthood, as well as slower functional improvement over time.

Impairments in functioning can involve various domains such as interpersonal relationships, occupational and vocational functioning, as well as level of autonomy^10,11^. Two recent meta-analyses have indicated that neglect seems to have a greater impact on functioning than abuse, and that socio-personal functioning is the domain most affected by CT in PD^3,4^. Although the mechanisms underlying the association between CT and functional disability remain poorly understood, the severity of symptoms likely plays a key role in this relationship^12^. Mediation analyses^13–16^ suggest that depressive symptoms partially or fully mediate the effects of CT on functioning. Additionally, Van Nierop et al.^17^ found that, regardless of psychiatric diagnosis, a history of trauma combined with a mix of psychotic and affective symptoms is associated with poorer role functioning and lower quality of life^17^.

Despite these advances, the number of studies exploring the relationship between CT, clinical symptoms, and functioning remains limited, and the ones that are available usually do not examine the interplay between different conditional effects. Furthermore, functioning has rarely been explored in relation to its various components and the way it is linked to specific traumatic events.

In recent years, network analysis has emerged as a novel, data-driven approach that is gaining significant attention in psychometrics. This approach conceptualizes symptoms, along with psychological, biological, and sociological components of diseases, as mutually interacting and often reciprocally reinforcing elements of a complex network^18^. These networks consist of nodes and edges. Nodes typically represent symptoms and external factors while edges indicate the conditional associations between them. Although network structures may be suggestive of causality, they do not establish causal relations directly. Instead, network models serve as exploratory tools for generating hypotheses^19,20^. They enable the examination of simultaneous relationships between variables without requiring prior assumptions about the direction of causality.

Several studies have used network analysis in populations with PD and schizotypy but also in the general population to explore the relationships between CT and clinical symptoms^21–25^ as well as between clinical symptoms and functioning^26–31^. These studies revealed that CT, clinical symptoms, and functioning are multidimensional constructs, interconnected through a complex web of associations. Specifically, they highlight numerous patterns linking various clinical symptoms to CT subtypes and domains of functioning.

To the best of our knowledge, only one study has examined the simultaneous interplay between CT, clinical symptoms, and functional impairment using network analysis^32^. This study used item-level scores for depressive and psychotic symptoms alongside total scores for trauma, depression-related impairment, and psychosis-related impairment. It found that trauma acted as a key bridging node linking depression and psychosis symptoms but did not reveal any significant connections between trauma and either depression or psychosis-related functional impairment. However, the study was conducted with a general population sample, using subthreshold psychotic symptoms. Additionally, it did not differentiate between childhood and adult trauma, which limits the ability to interpret causality, as trauma may result from symptoms rather than precede them^33^. Furthermore, the use of total scores for trauma and functional impairment does not capture the granular relationships between trauma subtypes and distinct functional domains.

This study has two primary objectives. The first is to explore the structure of a network that includes nodes related to CT, clinical symptoms, and functioning domains in individuals with FEP, at the time of initial treatment in an early intervention service (EIS), using item-level data. The first few years following a FEP are often considered a critical window for intervention. Gaining a deeper understanding of the interactions between clinical symptoms and biopsychosocial factors early in treatment, when medication confounding is minimal, may help inform the design of more personalized interventions. Accordingly, the second objective is to identify key nodes within the network that play a significant role in information transmission and could serve as potential targets for therapeutic interventions.

## Results

A total of 277 patients participated in this study. A total of 207 patients had been assessed at 2 months, and the remaining 70 at 6 months of follow-up. The sample consisted of 172 men and 105 women, with a mean age of 24.81 years. The mean duration of untreated psychosis was 420.09 days. Regarding treatment, 101 patients (36.46%) were receiving medication with a second- or third-generation antipsychotic. Of these, 56 patients were fully compliant with their medication, while 45 were partially compliant. The mean duration of antipsychotic medication at the time of evaluation was 5.97 months. Patients’ demographic and clinical characteristics are summarized in Table 1.

**Table 1:** Demographic and clinical characteristics of the study sample.

Variables	Study sample (*n* = 277)
Gender, *n* (%)	
Female	105 (37.9)
Male	172 (62.1)
Age (years), Mean (SD)	24.81 (5.03)
Marital status, *n* (%)	
Single	227 (81.94)
Married	25 (9.00)
Divorced	7 (2.52)
Cohabitation	15 (5.41)
Completed post-school training, *n* (%)	
Apprenticeship	71 (25.63)
Vocational school	16 (5.77)
University	36 (12.99)
Duration of untreated psychosis (days), Mean (SD)	420.09 (881.43)
Medication, *n* (%)	
No medication	157 (56.67)
Partial medication	45 (16.24)
Complete medication	56 (20.21)
Cumulative duration of antipsychotic treatment (months), Mean (SD)	5.97 (5.23)
PANSS, Mean (SD)	
PANSS positive factor	1.90 (1.24)
PANSS negative factor	2.31 (1.41)
PANSS general psychopathology	2.17 (1.33)
PANSS total score	2.14 (1.33)
aPAS, Mean (SD)	
Work status	4.87 (1.96)
Independence	2.27 (2.28)
Global functioning	3.52 (1.14)
Social-personal functioning	3.57 (1.28)
Interest	3.16 (1.32)
Energy	3.44 (1.32)
aPAS total score	3.47 (1.55)
Childhood trauma, *n* (%)	
Sexual abuse	44 (15.88)
Physical abuse	72 (25.99)
Emotional abuse	23 (8.30)
Emotional negligence	45 (16.24)
Physical negligence	10 (3.61)

*PANSS* Positive and Negative Syndrome Scale, *aPAS* Adapted version of the General Subscale of the Premorbid Adjustment Scale.

**Fig. 1:**
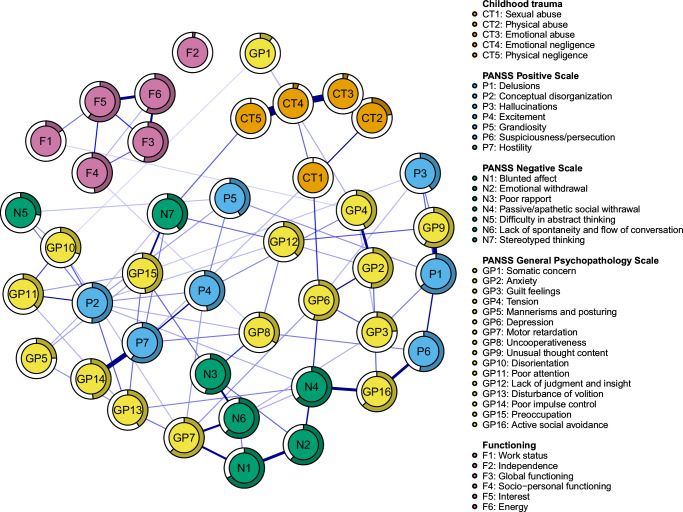
Network depicting the five subtypes of childhood trauma (sexual abuse, physical abuse, emotional abuse, emotional neglect, and physical neglect), the symptoms from the three subscales (positive, negative, and general psychopathology) of the Positive and Negative Syndrome Scale, and six domains of functioning (work status, independence, global functioning, socio-personal functioning, interest, and energy). Edges represent the unique associations between two variables, after conditioning on all other variables in the network. The thickness of each edge corresponds to the strength of the association.

The estimated network is depicted in Fig. 1. It contained 82 non-zero edges. Edge weights are reported in the Supplementary Table 1. It was a globally well-connected network, with only one isolated node—functioning’s node F2 (Independence). The clinical symptoms formed a dense complex whereas the CT types and functioning domains formed two highly connected clusters that were located at the periphery of the symptom complex and had few connections with it.

### Node centrality and predictability

The items with the highest node strength were, in descending order, CT4 (Emotional Neglect), CT3 (Emotional Abuse), P2 (Conceptual Disorganization), N4 (Passive/apathetic social withdrawal), P1 (Delusions), and CT2 (Physical Abuse). In terms of bridge strength, we identified CT1 (Sexual Abuse) and CT5 (Physical Neglect) among the trauma-related nodes, as well as F1 (Work Status) and F4 (Socio-personal Functioning) among the functioning-related nodes as important bridging nodes. Among the clinical symptoms, GP6 (Depression) was the node with the highest bridge strength, while P7 (Hostility), N7 (Stereotyped Thinking), GP4 (Tension), and GP8 (Uncooperativeness) were also identified as important bridging nodes (Supplementary Tables 2, 3 and Supplementary Figs. 1, 2).

The average node predictability was 0.40. With the exception of nodes F1 (Work Status) and F2 (Independence), whose predictability was very low, the other functioning-related nodes demonstrated above-average predictability, ranging from 0.47 for F4 (Socio-personal Functioning) to 0.60 for F5 (Interest). Predictability values for each node are presented in Supplementary Table 4 and are visually represented as rings surrounding each node in the main plot (Fig. 1).

### Shortest pathways

We constructed five networks to represent the shortest paths between each of the five CT nodes and all functioning nodes. The shortest paths from different CTs to the same functioning domain were generally quite similar, with all pathways involving clinical symptom nodes. However, some paths also included other CT nodes as well as functioning nodes other than the final target. When comparing the shortest paths in terms of the clinical symptoms they contained, we observed only three variations.

The first variation, found mostly in the shortest pathways from CT nodes to node F1 (Work Status), included the symptoms GP6 (Depression), GP2 (Anxiety), and GP4 (Tension). For instance, the shortest pathway from node CT3 (Emotional Abuse) to node F1 (Work Status) passed via the nodes CT2 (Physical Abuse), CT1 (Sexual Abuse), GP6 (Depression), GP2 (Anxiety), and GP4 (Tension).

The second variation appeared in the shortest pathways from most trauma nodes to nodes F3 (Global Functioning), F4 (Socio-personal Functioning), F5 (Interest), and F6 (Energy) and comprised nodes N7 (Stereotyped Thinking), GP12 (Lack of Judgment and Insight), and GP8 (Uncooperativeness). For example, the shortest pathway from node CT3 (Emotional Abuse) to F6 (Energy) passed via nodes CT4 (Emotional Neglect), CT5 (Physical Neglect), N7 (Stereotyped Thinking), GP12 (Lack of Judgment and Insight), GP8 (Uncooperativeness), F4 (Socio-personal Functioning), and F5 (Interest).

**Fig. 2:**
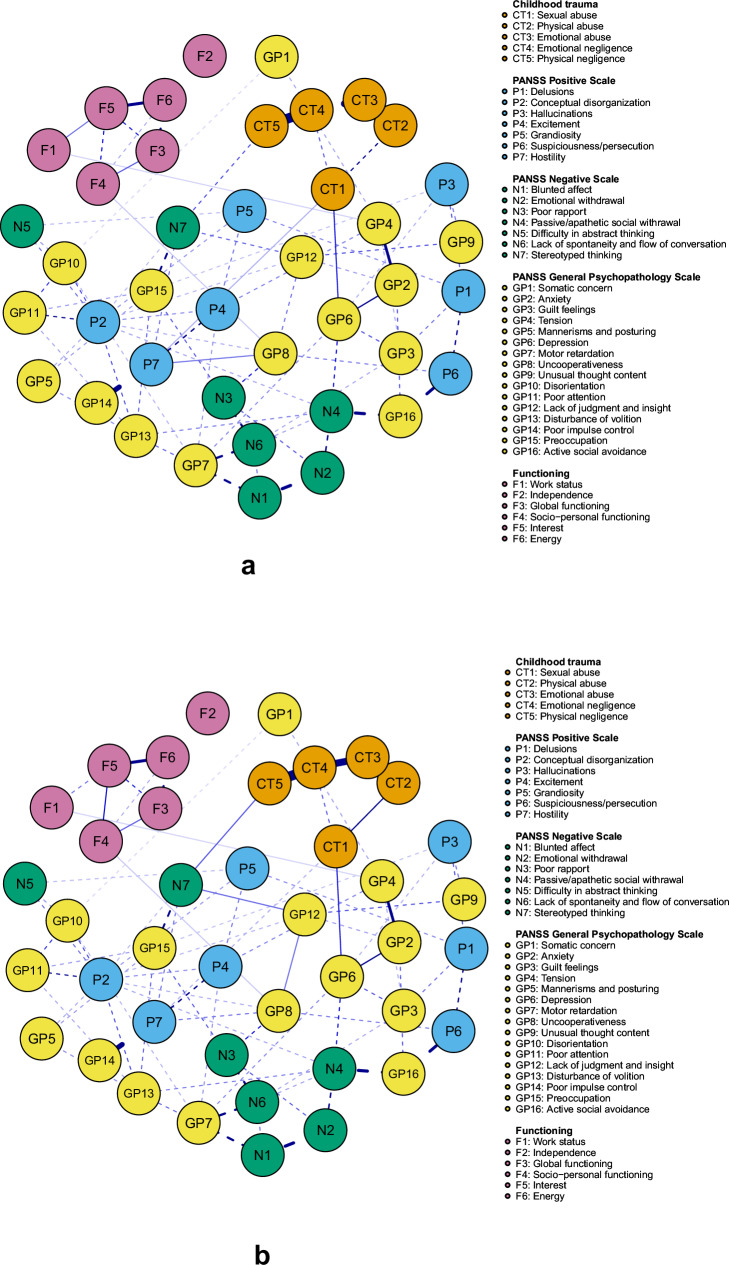
Shortest path networks linking CT to functioning. **a** Network depicting the shortest paths between sexual abuse (node CT1) and five domains of functioning (nodes F1, F3, F4, F5, F6); **b** Network depicting the shortest paths between emotional abuse (node CT3) and the same five domains of functioning. Solid lines indicate strong connections, while dashed lines represent connections within the network that are less relevant when analyzing shortest paths.

The third variation only connected CT1 (Sexual Abuse) to F3 (Global Functioning) and F4 (Socio-personal Functioning). Thus, the shortest pathway from CT1 to F3 passed via P4 (Excitement), P7 (Hostility), GP8 (Uncooperativeness), and F4 (Socio-personal Functioning). Figure 2 depicts the networks representing the shortest pathways from CT1 and CT3 to the functioning nodes. Networks with the shortest pathways for the other trauma nodes can be found in Supplementary Fig. 3.

### Stability, precision, and interpretability

The bootstrapped confidence intervals for edge weights in the estimated network, along with the results of the case-drop bootstrap and the bootstrapped difference tests for edge weights and node strength are included in the Supplementary Material (Supplementary Figs. 4–6). Most bootstrapped confidence intervals were very narrow, suggesting an overall stable network. However, the correlation stability coefficient for node strength was 0.21, falling below the recommended threshold of 0.25, which suggests limited estimation accuracy for node strength^34^.

## Discussion

This study estimated and analyzed a network incorporating CT subtypes, clinical symptoms, and various functional domains in a cohort of FEP patients during the very early stage of their treatment in an EIS. To the best of our knowledge, this is the first study to apply network analysis to a psychosis sample, combining CT data with clinical features and functional outcomes within the same network. There were three key findings: (1) different types of CT were linked to functioning via clinical symptoms but also via other forms of trauma; (2) certain domains of functioning formed a highly interconnected cluster, with few connections to the rest of the network; (3) two main shortest pathways connected CT to functioning, with each path showing distinct associations with various types of CT and functional domains.

Stability tests showed that the results of the estimation of edge weights were stable. However, the estimation of node strength exhibited limited accuracy. We will thus refrain from interpreting these results.

Within a pathway linking one CT type to functioning, other trauma types, aside from the starting node, were usually involved, with sexual abuse and physical neglect serving as gateways from the trauma cluster to the rest of the network. From a network analysis perspective, this suggests that the coexistence of multiple CT types opens the way to multiple or shorter pathways leading to functioning, thereby amplifying their effect. This connection pattern was also found in network analyses investigating the relationship between CT and symptoms^21,22,25^, and can be interpreted as evidence of a cumulative effect, where a higher number of trauma experiences is linked to poorer functioning^6^.

The functioning nodes were interconnected and connected as well to clinical symptoms, except for the independence level node, which was isolated, indicating no conditional association of this dimension with the rest of the network’s nodes. This finding aligns with a recent meta-analysis showing no association between CT and independent living in patients with psychotic disorders^3^. Regarding the other functioning nodes, four of them—global functioning, socio-personal functioning, interest, and energy—formed a highly connected cluster. Such a structure may present a hysteresis effect^35^. This means that once this structure shifts to an alternative state due to an external influence, (in this case, social dysfunction driven by clinical symptoms), it tends to remain in that state even after the external influence diminishes. This mode of information transmission is consistent with Velligan’s maintenance loop theory^36^, which suggests that decreased exposure to situations leads to decreased interest, fewer opportunities for reinforcement from the environment, and finally atrophy of social skills.

The shortest pathways analysis identified two primary routes linking CT to functioning. The first pathway, which linked all CT types to work status, had sexual abuse and depression as major bridging nodes. These results align with other studies in early psychosis, which show that depressive symptoms mediate the effect of childhood adversity on functioning in general^13,16^ as well as on occupational functioning in particular^15^. Furthermore, a recent network analysis of patients with schizophrenia spectrum disorders revealed that sexual abuse was connected to various clinical symptoms through the PANSS depressive factor^24^. The other important shortest pathway, linking most CT types to global functioning, socio-personal functioning, interest, and energy, involved physical neglect and stereotyped thinking as major bridging nodes. If stereotyped thinking is considered part of the PANSS disorganized factor in the five-factor model^37^, this result is in line with a recent network analysis on schizotypic patients, which identified physical neglect as being most strongly associated with disorganized schizotypy^23^.

Myin-Germeys and Van Os propose that in psychosis, there are two different pathways leading to different psychopathological expressions: an affective pathway associated with altered stress-sensitivity and leading to positive syndrome, non-deficit type I schizophrenia, and a cognitive pathway associated with cognitive deficits and leading to negative syndrome, deficit type II schizophrenia^38^. The two major pathways identified in this study seem to align with this hypothesis. The first pathway, which corresponds to Van Os’s affective pathway, had depression as its main bridging node, which is linked in the literature to stress sensitivity^39^. The second pathway, which could be linked to Van Os’s cognitive pathway, featured stereotyped thinking as its primary bridging node. Stereotyped thinking is a component of negative thought disorder whose presence in new-onset psychosis has been associated with evolution towards a chronic and deficit form of the disease^40^.

Additionally, the affective pathway was associated in our results with sexual abuse and served as the primary pathway leading to occupational functioning. In contrast, the cognitive pathway was linked to physical neglect and predominantly led to deficits in interpersonal functioning, interest, and energy. This differential association between sexual abuse and physical neglect with the two pathways aligns with findings from studies on neural development, which suggest that childhood abuse impacts emotional regulation capacities, while neglect impairs cognitive abilities^41^.

A conceptual framework that helps contextualize our findings is that of Teicher et al.^42^, who introduced the term “ecophenotype” to describe a phenotypic specialization resulting from environmental experience. Based on the results of this study, we could hypothesize that two distinct ecophenotypes may underlie the association between childhood trauma, clinical symptoms, and functioning in new-onset psychosis: an affective ecophenotype, strongly associated to sexual abuse and primarily affecting occupational functioning, and a cognitive ecophenotype, strongly linked to physical neglect and primarily impacting socio-personal functioning.

These findings have several implications for clinical practice. Considering not only the severity but also the number of different types of adversity a child has experienced may help identify vulnerable individuals who could be priority candidates for intervention^43^. Treatment of new-onset psychosis should not be limited to treating positive symptoms such as hallucinations and delusions but should also include the comprehensive evaluation and treatment of several other symptoms. Among these, depression—often inadequately treated in patients with psychosis^44^—should be a priority target. Moreover, if the hypothesis of two distinct ecophenotypes that differentially impact functioning is confirmed, it could pave the way for more personalized treatment approaches, with, for example, some patients benefiting more from cognitive remediation and others from coping techniques for dealing with the stresses of daily life^38^. Finally, our results show that interactions among different functional domains could potentially affect functioning beyond the influence of clinical symptoms alone. This highlights the importance of psychiatric rehabilitation interventions that specifically target several domains of functioning^45^.

### Limitations

This study has several limitations. A key limitation is the use of a tailored scale to assess childhood trauma rather than a widely validated instrument. This decision was made to better accommodate the specific characteristics of our study population and the study’s naturalistic design, where data collection was integrated into routine clinical practice. While this approach offers advantages—such as greater flexibility, reduced participant burden, more detailed data collection, and a lower risk of recall bias due to the trusting relationships built over the three-year follow-up period rather than a single time-point evaluation—it also has drawbacks. The absence of a standardized, evidence-based measure may limit comparability with previous studies and introduce potential measurement bias. Although the tailored scale was designed to capture relevant trauma-related experiences, its psychometric properties have not been formally established. Future research should replicate these findings using validated trauma assessment tools to strengthen the robustness and generalizability of the results.

Another limitation of this study is its cross-sectional design. While shortest path analysis can be used to explore predictive relationships and generate putative causal insights, these findings remain speculative. This study does not allow for definitive conclusions about causality, as it captures associations at a single point in time without providing evidence of the directionality or temporal sequence of effects. Therefore, although our study suggests potential causal pathways, these pathways are based on existing hypotheses from the literature. As a result, these findings should be considered exploratory. To test these hypotheses more rigorously, future research should involve well-powered prospective studies. These studies should collect data at multiple time points, with sufficient intervals between measurements to capture the dynamic relationship between clinical features and CT, and to better understand the direction of effects during the early stages of psychosis.

Moreover, the impact of trauma on mental health is highly complex and influenced by numerous factors that were not included in this study, such as the age of trauma exposure^46,47^, its duration and chronicity, substance abuse and genetic predisposition to psychosis^48^. Additionally, socioeconomic status^2^, social environment, and broader social determinants of health play crucial roles in shaping psychiatric outcomes. While this study focuses on clinical symptoms as potential mediators between CT and functioning, other mechanisms—such as attachment styles, structural brain changes, and neurocognitive or social cognitive impairments^12^—may also be critical in this relationship. Some of these factors were not included because they require different methodological approaches, such as twin studies, polygenic risk scores, longitudinal designs, or large-scale epidemiological research. Others were excluded due to the inherent constraints of network analysis, which necessitates a careful balance between the number of nodes and total sample size to ensure stable and interpretable results. As a result, this study adopted a relatively narrow focus. Although it does not encompass all the intervening factors in the relationship between CT and functioning, this study provides a targeted, symptom-focused perspective that complements, rather than contradicts, broader etiological research. Future studies integrating these additional factors will be essential for developing a more comprehensive understanding of the pathways linking childhood trauma to mental health outcomes.

## Conclusions

Our study provides support to the hypothesis of the existence of two distinct pathways to psychosis—an affective pathway and a cognitive pathway—each associated with a different disease phenotype. Furthermore, it provides evidence that these two phenotypes may be differentially associated with types of childhood trauma and impact functioning in distinct ways. This study is part of a growing body of research studying how exposure to childhood maltreatment leads to distinct phenotypic expressions within conventional diagnostic boundaries. A richer understanding of this phenomenon could lead to more personalized treatment approaches.

## Methods

### Participants and procedure

For this study, we used data from patients enrolled in the Treatment and Early Intervention in Psychosis Program (TIPP), an early intervention service within the Department of Psychiatry, Lausanne University Hospital, in Switzerland.

Patients are included in the TIPP program if they have crossed the psychosis threshold, as defined by the Psychosis Threshold subscale of the Comprehensive Assessment of At-Risk Mental States (CAARMS)^49^, and if they have not received antipsychotic treatment for more than 6 months. Specifically, reaching the CAARMS’ psychosis threshold requires a score of 6 on the delusions item, a score of 5–6 on the perceptual abnormalities item, and a score of 6 on the disorganization item. Additionally, symptoms must have occurred at least 3–6 times per week for more than one hour, or daily, even if lasting less than one hour, for a minimum duration of 7 days. For some patients, TIPP represents their first contact with psychiatric services for psychotic symptoms, while others have been referred from community centers or hospitals where they have already received treatment. For patients who are remitted or partially remitted from their symptoms at the time of evaluation, symptoms at their peak severity are assessed retrospectively using information from the patient, other healthcare professionals, and medical records. The decision on whether the psychosis threshold has been crossed is made during a team meeting with senior staff members.

More specifically, the program’s inclusion criteria are as follows: (1) age between 18 and 35; (2) having crossed the psychosis threshold as defined by the “Psychosis threshold” subscale of the Comprehensive Assessment of At-Risk Mental States (CAARMS) Scale; (3) residing in the catchment area (Lausanne and surroundings; population about 300,000). Exclusion criteria include: (1) prior use of antipsychotic medication for more than 6 months; (2) psychosis related to acute intoxication or organic brain disease; (3) an intelligence quotient below 70^50^.

In the TIPP program, each patient is followed up by a psychiatrist and a case manager for a period of three years. The program offers an integrated biopsychosocial treatment encompassing psychotherapy, psycho-education, family support, cognitive assessment and remediation, social support, assistance with employment, psychological interventions for cannabis use, and pharmacological treatment. A specially designed questionnaire is completed for all enrolled patients by their case managers. This tool assesses demographic characteristics, medical history, exposure to life events as well as symptoms and functioning. The information is gathered from patients and their families during the initial weeks of treatment and can be updated throughout follow-up as new information emerges. During follow-up assessments, a research psychologist assesses psychopathology, while the patient’s case manager assesses functioning as well as other aspects of treatment and comorbidities such as the insight level, treatment adherence, substance use, exposure to trauma, suicide attempts, and forensic events^13^.

Access to the TIPP clinical data for research purposes is granted under approval from the Human Research Ethics Committee of the Canton of Vaud (CER-VD; protocol #2020-00272). Participation in the TIPP program automatically includes consent for the use of clinical data in research, unless a patient explicitly opts out. To date, only four patients have declined permission for their clinical data to be used for research purposes.

### Measures and data collection

#### Childhood trauma

Childhood trauma was assessed using a specifically developed scale that evaluates the presence (yes/no) of 12 distinct types of trauma and records the age of exposure. Details about this instrument are provided in the Supplementary Material (Supplementary Note 1). Data on exposure to traumatic experiences were collected by trained case managers through a comprehensive evaluation process. This assessment was not limited to a single interview, questionnaire, or self-report but was instead embedded within a trusting relationship built progressively over the course of the treatment period. Case managers met with patients frequently, gathering detailed knowledge of their histories. With the patient’s consent and when deemed appropriate, additional information was collected from family members. In cases where discrepancies arose between the patient’s and family’s accounts or where uncertainty existed regarding the nature or timing of the trauma, the patient was excluded from the study to ensure data reliability.

Five types of trauma were included in this study: sexual abuse, emotional abuse, physical abuse, emotional neglect, and physical neglect. These are considered the five most prevalent types of trauma occurring during childhood. Sexual abuse refers to sexual molestation and/or rape. Physical abuse includes physical attack or assault, or being repetitively beaten by parents, relatives, or caregivers. Emotional abuse is defined as verbal assaults on a child’s sense of worth or well-being, or any humiliating or demeaning behavior directed toward a child by an adult or older person. Physical neglect involves the failure of caretakers to meet a child’s basic physical needs, including food, shelter, clothing, safety, and health care. Emotional neglect is defined as the failure of caretakers to meet child’s basic emotional and psychological needs, including love, belonging, nurturance, and support^51^. We included any trauma occurred before the age of 16, as trauma occurring after this age may coincide with the prodromal phase of a first psychotic episode^52^.

#### Clinical symptoms and functioning

To assess clinical symptoms, we utilized items from the Positive and Negative Syndrome Scale (PANSS)^53^, a clinician-rated tool comprising 30 items divided into three subscales: positive symptoms, negative symptoms, and general psychopathology. Each item is scored on a 7-point Likert scale, ranging from 1 (absent) to 7 (very severe). In our sample, the PANSS demonstrated strong internal consistency, with a Cronbach’s alpha of 0.87.

Functional impairment and its domains were measured using an adapted version of the “General Subscale” of the Premorbid Adjustment Scale (PAS) (Supplementary Note 2). The original PAS “General Subscale” was designed to assess the highest level of functioning achieved prior to the onset of illness^54^. It includes nine clinician-rated items: 1. Education; 2. Duration of paid employment; 3. Abruptness of job change; 4. Frequency of job change; 5. Establishment of independence; 6. Global assessment of functioning; 7. Social-personal adjustment; 8. Degree of interest in life; 9. Energy level. Each item is scored on a 7-point Likert scale, with 0 representing the healthiest level of functioning and 6 representing the least healthy.

The PAS “General Subscale” can also be applied to assess functioning after the onset of psychosis, as it enables comparison between premorbid PAS scores and those obtained during follow-up. This comparison provides a framework for operationalizing functional recovery as the change in PAS scores over time^55^. In TIPP, we use an adapted version of the PAS “General Subscale” to evaluate functioning during the 2 months preceding each assessment. In this version, three items—education, abruptness of job change, and frequency of job change—were excluded, as they are not relevant for assessing functioning over such a short timeframe.

We chose to use item scores from the adapted PAS “General Subscale” rather than the global scores provided by other tools, such as the Global Assessment of Functioning (GAF) or the Social and Occupational Functioning Assessment Scale (SOFAS), which are also used in patient evaluations. The PAS offers greater granularity, providing detailed insights into specific subcomponents of functioning that are amalgamated into composite scores in these alternative instruments. This same approach to assess functioning has also been used in other studies^55,56^.

This study employed a cross-sectional design, focusing specifically on the patients’ initial assessment and including data on clinical symptoms and functioning at that time point. Data on symptom severity and functioning were collected during each patient’s first comprehensive evaluation after joining the TIPP program. For most patients, this evaluation occurred 2 months after enrollment. However, for a minority of patients who were highly symptomatic at the 2-month mark and unable to complete the full evaluation, the assessment was deferred to 6 months after the start of treatment. Importantly, data for both symptom severity and functioning were drawn from the same time point (either 2 or 6 months, depending on the patient’s condition).

### Statistical analysis

As the data consisted of both ordinal (PANSS and PAS) and binary (CT) data, a network was estimated using Mixed Graphical Models designed for mixed data^57^. Missing data (2.9%) were imputed using the k-nearest neighbors (KNN) method^58^, which replaces missing values with the mean value from the closest k nearest neighbors. The value of *k* was set to 5. Consistent with recent studies^22,59^, ordinal data were treated as continuous, and due to data skewness, a non-paranormal transformation^60^ was applied prior to network estimation. For network selection, L1-penalized regression (LASSO) was employed, and to prioritize sensitivity, cross-validation (CV) was used to select the penalty parameter. CV is less conservative than the Extended Bayesian Information Criterion (EBIC) commonly used in network analysis in psychometrics^61^. The number of folds was set to 10.

Node centrality, which reflects the importance of a node within the network, was examined by computing node strength^62^, as it is considered the most stable and interpretable among centrality measures^34^. Node strength is defined as the sum of the absolute edge weights connected to a node, indicating the strength of its interactions with other nodes^62^. A higher node strength suggests greater centrality, meaning the variable exerts more influence within the network. Key nodes linking CT subtypes with domains of functioning were identified by computing the bridge strength, which quantifies how strongly a node connects different communities within the network. Nodes with high bridge strength serve as intermediaries, facilitating interactions between otherwise distinct clusters of variables. In this study, three communities were defined: CT, clinical symptoms, and functioning. Bridge strength was calculated as the sum of edge weights connecting a node to nodes in other communities^63^.

The shortest paths from each CT node to each functioning node were computed using Dijkstra’s algorithm^64^. Shortest path analysis identifies the most direct and efficient relationships between two nodes based on the strength of the connections linking them. Since the partial correlations between a node and all other nodes in the network are directly related to the regression coefficients in a multiple regression model, these correlations can be interpreted as predictive effects. The chain of nodes along the shortest path represents the most efficient route, mediating the predictive relationship between the two nodes^21^. Additionally, given the multifactorial nature of functional impairment in psychotic patients, node predictability, which reflects the extent to which the variance of each node is explained by the factors included in the network^65^, was analyzed.

The stability, precision, and interpretability of the results were analyzed by computing bootstrapped confidence intervals, as well as by computing correlation stability coefficients and performing bootstrapped difference tests for edges and node strength^34^.

All analyses were performed using R statistical software version 4.4.1^66^. For network estimation and predictability computation, the mgm package^57^ was used. The qgraph package^67^ was employed for network visualization as well as shortest pathways computation and visualization while the bootnet package^34^ was used for stability analysis.

## Supplementary information

Supplementary Material

## Data Availability

The data underlying this study involve research with human subjects and are subject to legal restrictions under Swiss regulations to ensure confidentiality. As a result, the data cannot be publicly shared. However, they may be made available upon reasonable request to the corresponding author, provided such requests comply with applicable ethical guidelines and legal requirements.

## Code availability

All analyses were performed using R statistical software version 4.4.1. The code used for data analysis is available upon request from the corresponding author.
