# Supplementary material for: Pathways between childhood trauma, clinical symptoms, and functioning in new-onset psychosis: novel insights from a network analysis approach
Source: Schizophrenia (Heidelb). 2025 May 15;11(1):75. doi: 10.1038/s41537-025-00620-2 (PMC12081628; doi:10.1038/s41537-025-00620-2)
Supplement: Supplementary file 1 — Supplementary Material [file 41537_2025_620_MOESM1_ESM.pdf]

Supplementary Table 1.

Edge weights

**Edge**	**Weight**
CT1-CT2	0.25447638
CT1-CT4	0.16306795
CT1-P7	0.07830003
CT1-GP6	0.23389338
CT2-CT3	0.90776116
CT3-CT4	0.63763802
CT4-CT5	0.83069570
CT5-N7	0.16994434
P1-P3	0.17387295
P1-P5	0.11562408
P1-P6	0.26584572
P1-GP3	0.13847440
P1-GP9	0.48697670
P2-P4	0.11266669
P2-P5	0.10059197
P2-N4	0.08761517
P2-N5	0.13863960

P2-N7	0.06608784
P2-GP5	0.07947203
P2-GP8	0.07995176
P2-GP10	0.17532280
P2-GP11	0.25189711
P2-GP12	0.07212099
P2-GP13	0.17315472
P3-GP6	0.07525100
P3-GP9	0.13932972
P3-GP15	0.06037232
P4-P5	0.13757144
P4-P7	0.26738692
P4-GP4	0.12202281
P4-GP7	0.10607462
P5-N5	0.06700654
P6-GP8	0.13070740
P6-GP16	0.37051485
P7-N7	0.13937510
P7-GP8	0.13883436
P7-GP14	0.50780944

N1-N2	0.38293726
N1-N6	0.13898311
N1-GP7	0.31977921
N2-N3	0.16113881
N2-N4	0.27476041
N3-N6	0.31519846
N3-GP8	0.23738035
N3-GP15	0.16270992
N4-N6	0.08945928
N4-GP6	0.25101348
N4-GP13	0.12844029
N4-GP16	0.38248532
N5-GP10	0.09926298
N6-GP3	0.08430779
N6-GP7	0.32443766
N7-GP12	0.15306933
N7-GP15	0.30007033
GP1-GP4	0.08804433
GP1-GP10	0.03769558
GP2-GP4	0.35520197

GP2-GP6	0.25752520
GP2-GP12	0.09436190
GP2-GP16	0.17003056
GP3-GP4	0.08137536
GP3-GP6	0.16991502
GP4-F1	0.04447777
GP5-GP13	0.10938285
GP5-GP15	0.09713516
GP6-GP7	0.11433503
GP7-GP10	0.07178468
GP7-GP13	0.14973486
GP8-GP12	0.14368326
GP8-F4	0.05320538
GP9-GP12	0.17345400
GP10-GP11	0.19412010
GP11-GP13	0.08327621
GP11-GP15	0.09910015
GP13-GP15	0.15576788
F3-F4	0.16255298
F3-F5	0.22179235

F3-F6	0.31959362
F4-F5	0.24837013
F5-F6	0.37260559
F1-F5	0.13666722
F4-F6	0.07907916

Supplementary Table 2.

Absolute strength of each node in the network

Node	Strength
Sexual abuse	0.7297377
Physical abuse	1.1622375
Emotional abuse	1.5453992
Emotional neglect	1.6314017
Physical neglect	1.0006400
Delusions	1.1807938
Conceptual disorganisation	1.3375207
Hallucinations	0.4488260
Excitement	0.7457225
Grandiosity	0.4207940
Suspiciousness/persecution	0.7670680
Hostility	1.1317059
Blunted affect	0.8416996
Emotional withdrawal	0.8188365
Poor rapport	0.8764275
Passive/apathetic social withdrawal	1.2137740
Difficulty in abstract thinking	0.3049091
Lack of spontaneity and flow of conversation	0.9523863
Stereotyped thinking	0.8285469

Somatic concern	0.1257399
Anxiety	0.8771196
Guilt feelings	0.4740726
Tension	0.6911222
Mannierims and posturing	0.2859900
Depression	1.1019331
Motor retardation	1.0861461
Uncooperativeness	0.7837625
Unusual thought content	0.7997604
Disorientation	0.5781861
Poor attention	0.6283936
Lack of judgement and insight	0.6366895
Disturbance of volition	0.7997568
Poor impulse control	0.5078094
Preoccupation	0.8751557
Active social avoidance	0.9230307
Work status	0.1811450
Independence	0.0000000
Global functioning	0.7039390
Socio-personal functioning	0.5432076
Interest	0.9794353
Energy	0.7712784

Supplementary Figure 1. Line plot depicting the node strength across all network's nodes, arranged vertically along the y-axis, with strength values represented along the x-axis. (CT1: Sexual abuse, CT2: Physical abuse, CT3: Emotional abuse, CT4: Emotional neglect, CT5: Physical neglect, P1: Delusions, P2: Conceptual disorganisation, P3: Hallucinations, P4: Excitement, P5: Grandiosity, P6: Suspiciousness/persecution, P7: Hostility, N1: Blunted affect, N2: Emotional withdrawal, N3: Poor rapport, N4: Passive/apathetic social withdrawal, N5: Difficulty in abstract thinking, N6: Lack of spontaneity and flow of conversation, N7: Stereotyped thinking, GP1: Somatic concern, GP2: Anxiety, GP3: Guilt feelings, GP4:

Tension, GP5: Mannierims and posturing, GP6: Depression, GP7: Motor retardation, GP8: Uncooperativeness, GP9: Unusual thought content, GP10: Disorientation, GP11: Poor attention, GP12: Lack of judgement and insight, GP13: Disturbance of volition, GP14: Poor impulse control, GP15: Preoccupation, GP16: Active social avoidance, F1: Work status, F2: Independence, F3: Global functioning, F4: Socio-personal functioning, F5: Interest, F6: Energy)

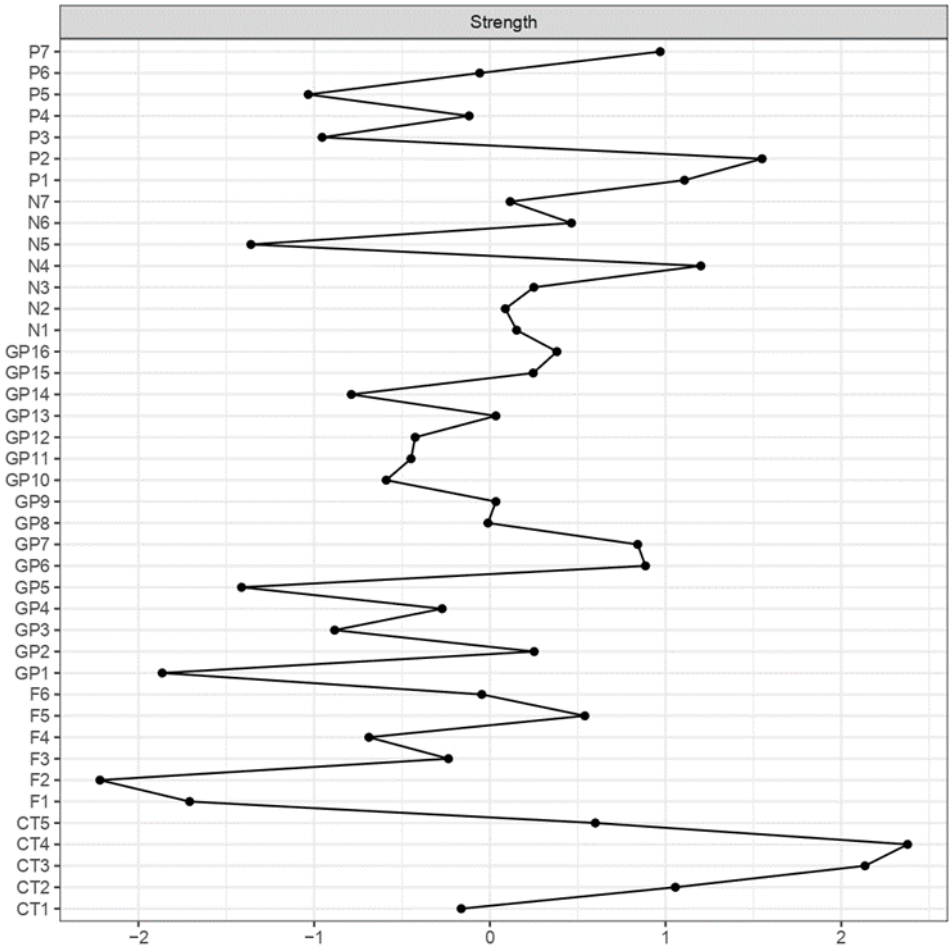

Supplementary Table 3.

Absolute values of bridge strength for the most important bridging nodes in the network

Node	Bridge strength
Sexual abuse	0.31219342

Physical neglect	0.16994434
Hostility	0.07830003
Stereotyped thinking	0.16994434
Tension	0.04447777
Depression	0.23389338
Uncooperativeness	0.05320538
Work status	0.04447777
Socio-personal functioning	0.05320538

Supplementary Figure 2.

Scatter plot depicting bridge strength values for each node in the network. Each node is represented by an index along the x-axis and its corresponding bridge strength on the y-axis. (1: Sexual abuse, 2: Physical abuse, 3: Emotional abuse, 4: Emotional neglect, 5: Physical neglect, 6: Delusions, 7: Conceptual disorganisation, 8: Hallucinations, 9: Excitement, 10: Grandiosity, 11: Suspiciousness/persecution, 12: Hostility, 13: Blunted affect, 14: Emotional withdrawal, 15: Poor rapport, 16: Passive/apathetic social withdrawal, 17: Difficulty in abstract thinking, 18: Lack of spontaneity and flow of conversation, 19: Stereotyped thinking, 20: Somatic concern, 21: Anxiety, 22: Guilt feelings, 23: Tension, 24: Mannierims and posturing, 25: Depression, 26: Motor retardation, 27: Uncooperativeness, 28: Unusual thought content, 29: Disorientation, 30: Poor attention, 31: Lack of judgement and insight, 32: Disturbance of volition, 33: Poor impulse control, 34: Preoccupation, 35: Active social avoidance, 36: Work status, 37: Independence, 38: Global functioning, 39: Socio-personal functioning, 40: Interest, 41: Energy)

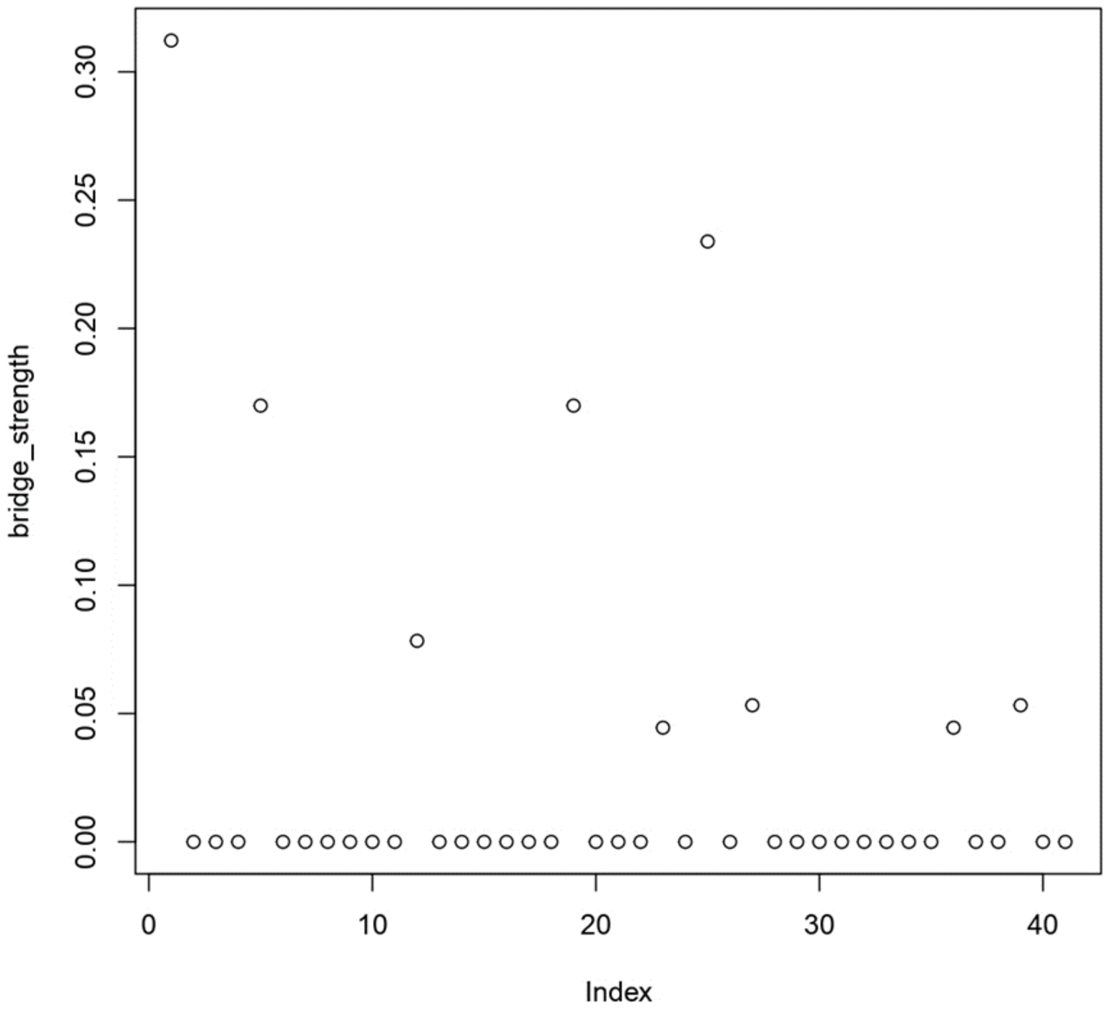

Supplementary Table 4.

Predictability of the network's nodes, defined as the proportion of each node's variance explained by its connections to other nodes

Nodes	Predictability
Sexual abuse	0.000
Physical abuse	0.222
Emotional abuse	0.043
Emotional neglect	0.044

Physical neglect	0.000
Delusions	0.639
Conceptual disorganisation	0.503
Hallucinations	0.401
Excitement	0.479
Grandiosity	0.413
Suspiciousness/persecution	0.533
Hostility	0.595
Blunted affect	0.665
Emotional withdrawal	0.611
Poor rapport	0.542
Passive/apathetic social withdrawal	0.640
Difficulty in abstract thinking	0.270
Lack of spontaneity and flow of conversation	0.619
Stereotyped thinking	0.396
Somatic concern	0.104
Anxiety	0.515
Guilt feelings	0.241
Tension	0.425
Mannierims and posturing	0.247
Depression	0.544
Motor retardation	0.622
Uncooperativeness	0.341
Unusual thought content	0.579
Disorientation	0.304
Poor attention	0.375
Lack of judgement and insight	0.376
Disturbance of volition	0.403
Poor impulse control	0.483
Preoccupation	0.476
Active social avoidance	0.626
Work status	0.156
Independence	0.021

Global functioning	0.534
Socio-personal functioning	0.479
Interest	0.601
Energy	0.571

Supplementary Figure 3. Networks depicting the shortest paths between Physical abuse (node CT2), Emotional neglect (CT4), Physical neglect (CT5) and five domains of functioning (nodes F1, F3, F4, F5, F6). Solid lines indicate strong connections, while dashed lines represent connections within the network that are less relevant when analyzing shortest paths.

a. Shortest paths between Physical abuse and domains of Functioning.

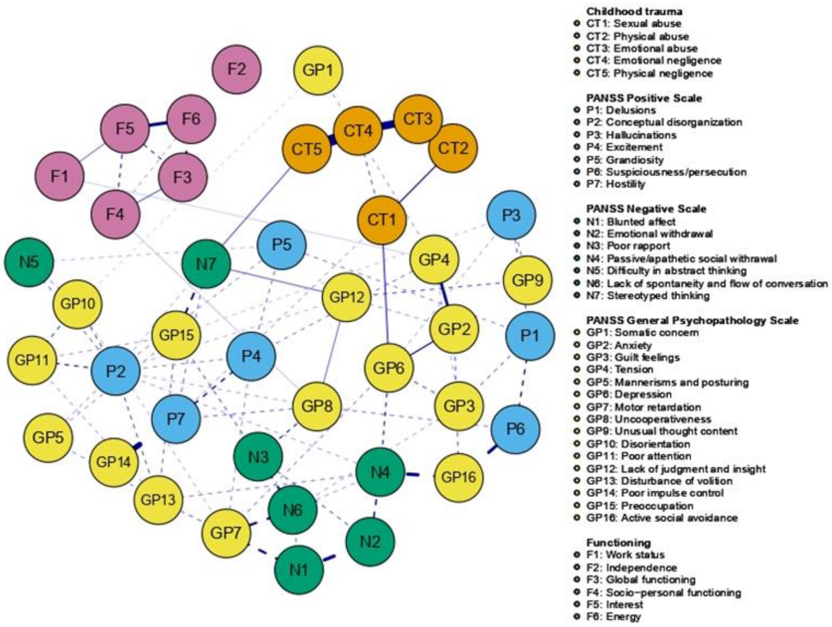

b. Shortest paths between Emotional negligence and domains of Functioning

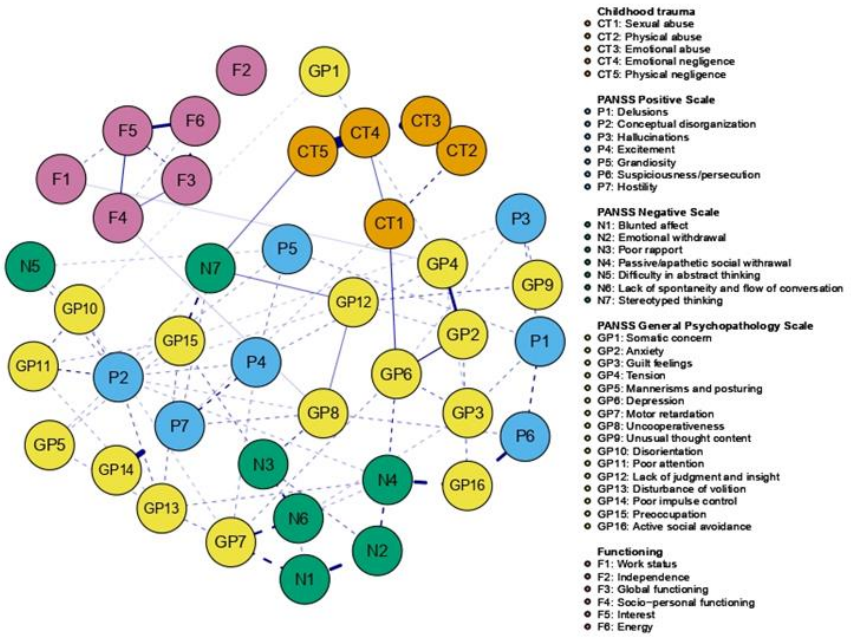

c. Shortest paths between Physical negligence and domains of Functioning

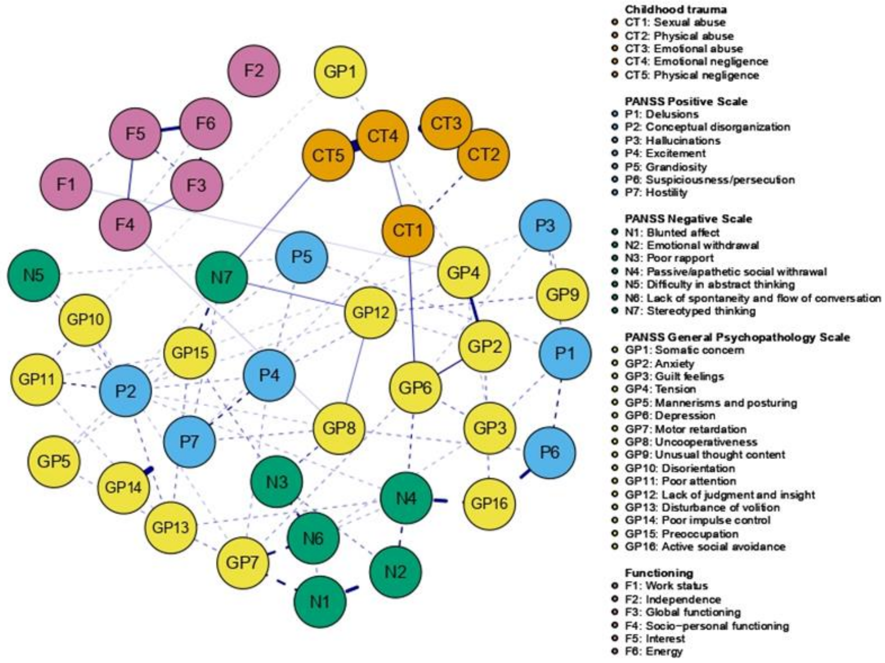

Supplementary Figure 4. Bootstrapped confidence intervals (CIs) of edge weights for the estimated network. Each horizontal line corresponds to an edge in the network, ordered from the highest to the lowest estimated edge weight. The gray area indicates the bootstrapped CIs. The y-axis labels have been removed for clarity.

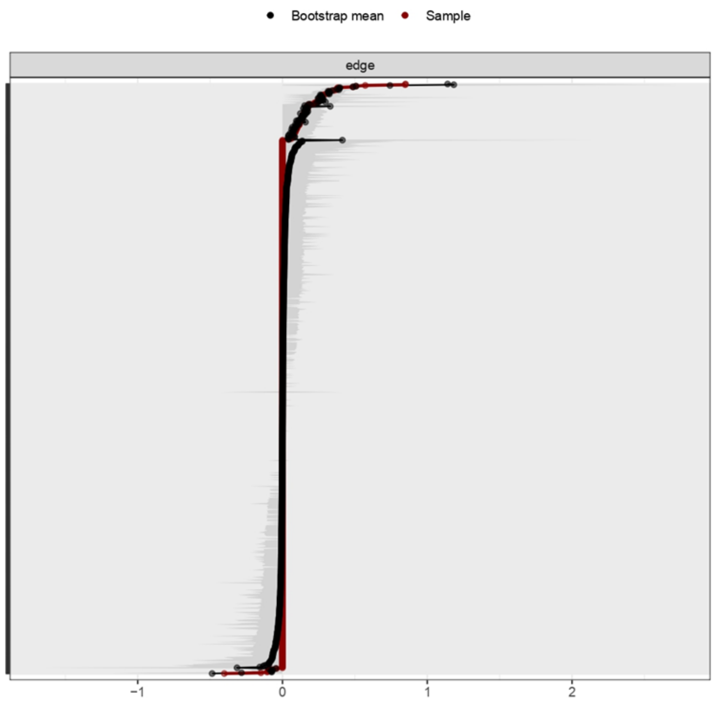

Supplementary Figure 5. Average correlations between node strength of networks sampled with persons dropped and the original sample. The line represents the mean correlation, while the shaded area indicates the range from the 2.5th to the 97.5th quantile.

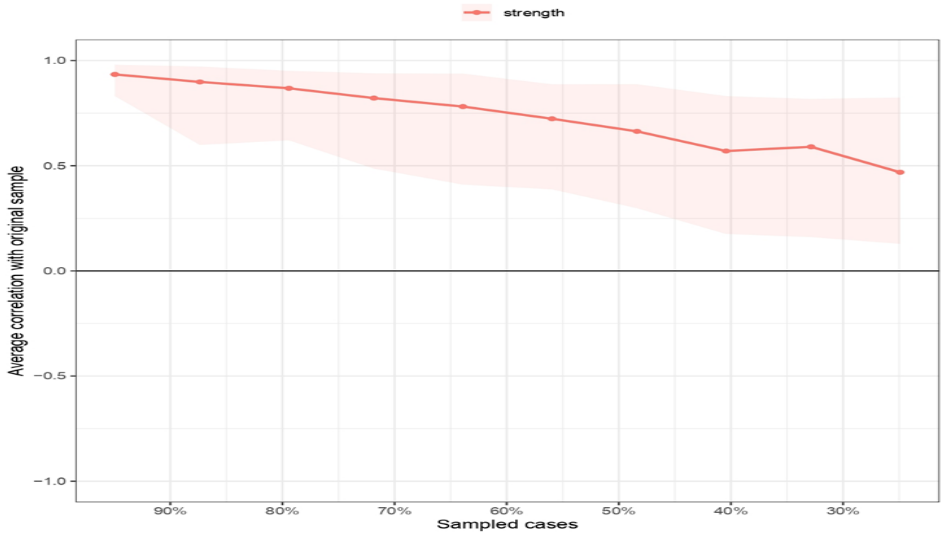

Supplementary Figure 6. Bootstrapped difference tests between edge weights (above) and node strength (below). Gray boxes indicate nodes or edges with no significant differences, while black boxes represent those that differ significantly. The depth of color of colored boxes in the edge-weight plot corresponds to the magnitude of the edge weights, while white boxes in the centrality plot display the values of node strength. (CT1: Sexual abuse, CT2: Physical abuse, CT3: Emotional abuse, CT4: Emotional neglect, CT5: Physical neglect, P1: Delusions, P2: Conceptual disorganisation, P3: Hallucinations, P4: Excitement, P5: Grandiosity, P6: Suspiciousness/persecution, P7: Hostility, N1: Blunted affect, N2: Emotional withdrawal, N3: Poor rapport, N4: Passive/apathetic social withdrawal, N5: Difficulty in abstract thinking, N6: Lack of spontaneity and flow of conversation, N7: Stereotyped thinking, GP1: Somatic concern, GP2: Anxiety, GP3: Guilt feelings, GP4: Tension, GP5: Mannierims and posturing, GP6: Depression, GP7: Motor retardation, GP8: Uncooperativeness, GP9: Unusual thought content, GP10: Disorientation, GP11: Poor attention, GP12: Lack of judgement and insight, GP13: Disturbance of volition, GP14: Poor impulse control, GP15: Preoccupation, GP16: Active social avoidance, F1: Work status, F2: Independence, F3: Global functioning, F4: Socio-personal functioning, F5: Interest, F6: Energy)

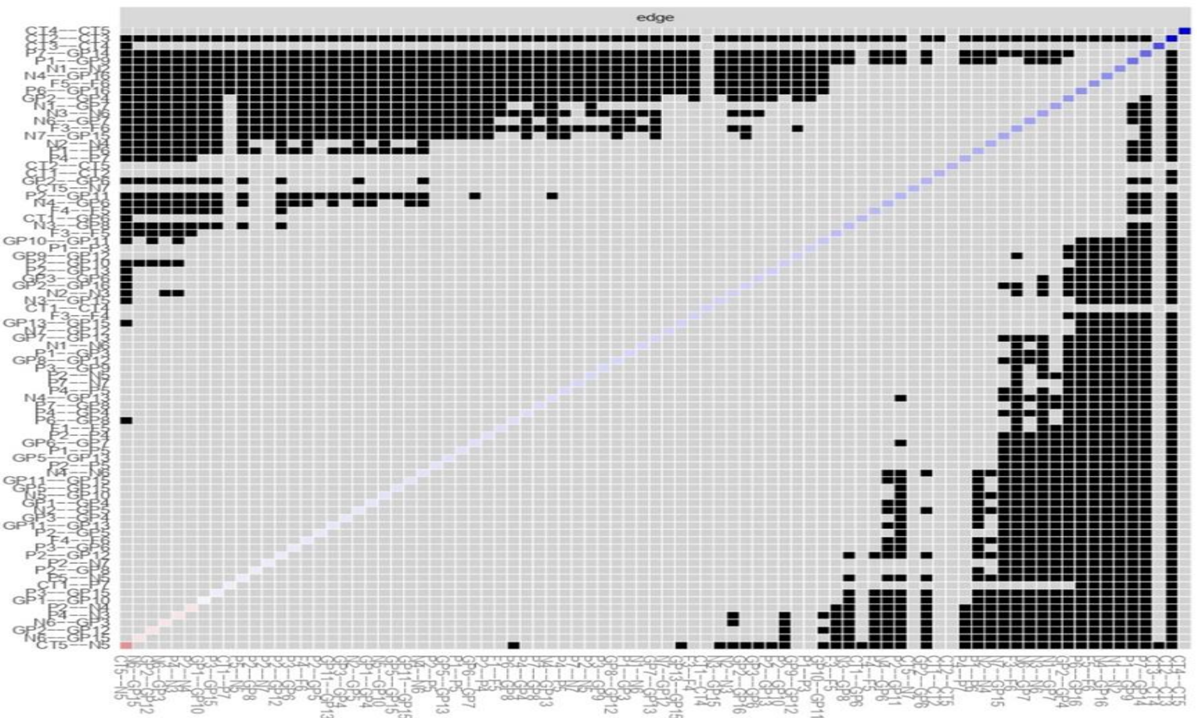

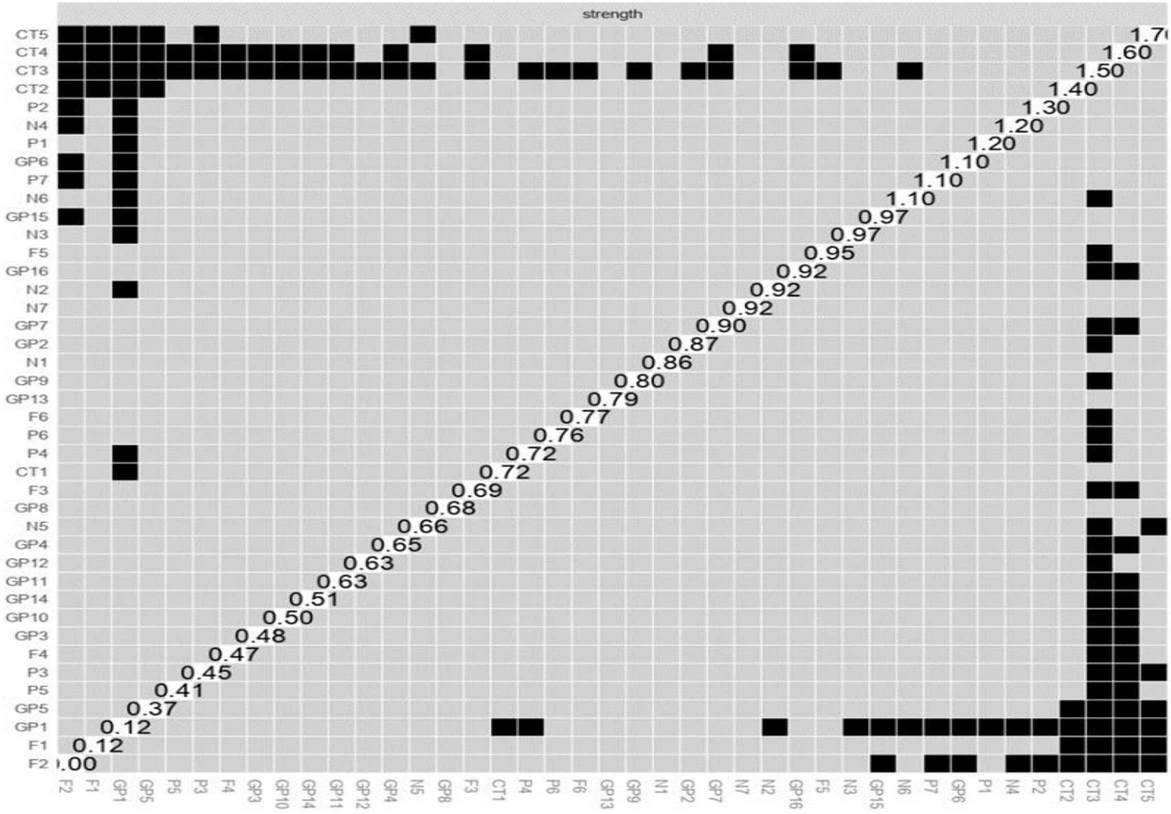

Supplementary Note 1.

The tailored scale employed in TIPP for assessing childhood trauma

Traumatic Events in the Past (and Temporal Relationship with Psychotic Episode)

Traumatic Events?

If yes, which one? (0=no, 1=yes)	Premorbid	Prodromal	Intrapsychic	Repeated	Age
Adoption	0 1	0 1	0 1	0 1	
Parental Separation	0 1	0 1	0 1	0 1	
Relationship Issues	0 1	0 1	0 1	0 1	
Sexual Abuse	0 1	0 1	0 1	0 1	
Physical Abuse	0 1	0 1	0 1	0 1	
Death of a Loved One	0 1	0 1	0 1	0 1	
Migration	0 1	0 1	0 1	0 1	
Emotional Neglect	0 1	0 1	0 1	0 1	
Emotional Abuse	0 1	0 1	0 1	0 1	
Physical Neglect	0 1	0 1	0 1	0 1	
Bullying	0 1	0 1	0 1	0 1	
Abandonment	0 1	0 1	0 1	0 1	
Other	0 1	0 1	0 1	0 1	

Supplementary Note 2.

An adapted version of the 'General Subscale' from the PAS scale, used in TIPP to evaluate the patient's functioning over the two months prior to assessment

A) In the last two months, patient was employed for pay or functioning in school

0 All the time.

1

2 Half the time.

3

4 Briefly, about 25 percent of the time.

5

6 Never

B) Establishment of independence

0 Successfully established residence away from family home, financially independent of parents.

2 Made unsuccessful attempts to establish independent residence, lives in parents' home, but pays parents room and board, otherwise financially independent.

4 Lives in parents' home, receiving an allowance from parents which patient budgets to pay for entertainment, clothes, etc.

6 Made no attempt to leave home or be financially independent.

C) Global assessment of highest level of functioning achieved in the last two months

0 Fully able to function success fully in and take pleasure from (1) school or job; (2) friends; (3) intimate sexual relationships; (4) church, hobbies, etc. Enjoys life and copes with it well.

2 Able to function well in and enjoys some spheres of life, but has a definite lack of success in at least one area.

4 Minimum success and pleasure in three areas of life.

6 Unable to function in or enjoy any aspect of life.

D) Social-personal adjustment (assess the situation over the past two months)

0 A leader or officer in formally designated groups, clubs, organizations, or athletic teams in senior high school, vocational school, college, or young adulthood. Involved in intimate, close relationship with others.

1 An active and interested participant, but did not play a leading role in groups of friends, clubs, organizations, or athletic teams, but was involved in close relationships with others also.

2 A nominal member, but had no involvement in or commitment to, groups of friends, clubs, organizations, etc. Had close relationships with a few friends.

3 From adolescence through early adulthood had a few casual friends.

4 From adolescence through early adulthood had no real friends, only superficial relationships.

5 From adolescence through early adulthood (i.e., after childhood), quiet, seclusive, preferred to be by self, minimal efforts to maintain any contact at all with others.

6 No desire to be with peers or others. Either asocial or antisocial.

E) Degree of interest in life

0 Keen, ambitious interest in some of the following: home, family, friends, work, sports, art, pets, gardening, social activities, music, and drama.

2 Moderate degree of interest in several activities including social gatherings, sports, music, and opposite sex.

4 Mild interest in a few things such as job, family, quiet social gatherings. The interest is barely sustaining.

6 Withdrawn and indifferent to ward life interests of average individual. No deep interests of any sort.

F) Energy level

0 Strong drive, keen, active, alert interest in life. Liked life and had energy enough to enjoy it. Outgoing and adequate in meeting life.

2 Moderately adequate drive, energy, interest, as described above.

4 Moderately inadequate energy level. Tended toward submissive, passive reactions. Showed some potential to face life's problems, but would rather avoid them than expend the necessary energy.

6 Submissive, inadequate, passive reactions. Weak grasp on life, does not go out to meet life's problems, does not participate actively, but passively accepts his lot without having the energy to help self.
